# Fertility after expanded polytetrafluoroethylene use after endometrioma cystectomy: a pilot study

**DOI:** 10.3389/frph.2023.1231029

**Published:** 2023-11-22

**Authors:** Patrick P. Yeung, Melody S. Su, John Voltz, Jeffrey A. Gavard

**Affiliations:** ^1^ Obstetrics, Gynecology, and Women’s Health, Saint Louis University School of Medicine, St. Louis, MO, United States; ^2^ Saint Louis University School of Medicine, St. Louis, MO, United States

**Keywords:** endometriosis, excision surgery, expanded polytetrafluoroethylene, infertility, pregnancy, pelvic adhesions

## Abstract

**Introduction:**

Pregnancy rates after the placement of expanded polytetrafluoroethylene (ePTFE, trade name Gore-Tex®) for adhesion prevention following cystectomy of endometriomas ≥3 cm and excision of endometriosis were analyzed in this pilot study.

**Methods:**

A prospective cohort study was performed at a single tertiary care center. 56 women qualified for the study and underwent surgery. Expanded polytetrafluoroethylene placement around affected ovaries was self-selected. Inclusion criteria for analysis were pathology-confirmed endometrioma ≥3 cm, no hysterectomy at time of surgery, ≥1 year of postoperative survey completion, and absence of strategies to avoid pregnancy. 18 women in the ePTFE group and 11 women in the control group met inclusion criteria for analysis. 16 of the 18 women in the ePTFE group and 7 of the 11 women in the control group were affected by infertility. Absolute pregnancy rates and cumulative 4-year pregnancy rates, which are based on survival analysis using lifetables and adjust for varying follow-up times, were calculated for all women as well as for women with infertility only.

**Results:**

High cumulative 4-year pregnancy rates were observed for women with expanded polytetrafluoroethylene compared to women without (85% vs. 65%, p = 0.69). High cumulative 4-year pregnancy rates for women with infertility prior to surgery were observed for women with expanded polytetrafluoroethylene compared to women without (83% vs. 33%, p = 0.89).

**Discussion:**

There are consistent trends, although not statistically significant, seen in pregnancy rates for women with ePTFE compared to women without, particularly in those with a history of infertility prior to ePTFE use. This is the first study examining how adhesion prevention strategy targeting the adnexa during surgery for endometriosis affects pregnancy rates. The trend towards increased pregnancy rates with expanded polytetrafluoroethylene use, particularly in patients with a history of infertility, is promising and warrants further study with larger groups.

## Introduction

Endometriosis is a chronic inflammatory condition characterized by endometrial tissue outside of the uterus. It commonly affects women with infertility, which the World Health Organization defines as the inability to conceive after 12 months of regular unprotected intercourse or after 6 months of fertility focused intercourse. While the etiology is not currently understood, the association between endometriosis and infertility has been shown as early as the 1980s (1).

The formation of pelvic adhesions secondary to endometriosis has been a proposed mechanism for this relationship to infertility. The tendency of endometriosis to recur means that affected women may undergo multiple surgeries, which further predisposes them to adhesion formation. Adhesions related to endometriosis are not only associated with a negative impact on quality of life (2) but also with infertility through anatomic distortion and subsequent disruption of normal tubal function (3–6). While adhesion prevention strategies with liquid and solid barrier agents in endometriosis have been explored and established in prior research, there are currently no studies that investigate adhesions in relation to clinically relevant endpoints such as pregnancy rate (4, 7). Fulfilling this research gap will allow for improved treatment options for women with endometriosis and infertility. This study focuses on pregnancy rates as a relevant clinical outcome in regards to adhesion prevention in endometriosis.

Endometriomas, also called chocolate cysts, are of particular concern in terms of infertility due to compromised ovarian reserve. These ovarian cysts composed of endometrial-like tissue filled with old blood affect up to 44% of women with endometriosis (5, 8). While there is evidence that endometriomas diminish ovarian reserve, the pathophysiology is currently unclear with a space-occupying effect and a direct toxic effect having both been proposed as possible mechanisms (6). The presence of bilateral ovarian cysts has been found to be a strong predictor of infertility, and the adnexa are the predominant sites of adhesion reformation after adhesiolysis (9). Multiple studies have demonstrated the benefit of cystectomy of endometriomas (10–13). Some data suggest, however, that cystectomy may lower ovarian reserve, which is reflected by a decrease in anti-Müllerian hormone and ovarian antral follicle count, thereby reducing fertility rates (14, 15). The 2022 European Society of Human Reproduction and Embryology (ESHRE) guideline recommends that the decision to pursue surgery for endometriosis-associated fertility be guided by patient symptoms, preferences, ovarian reserve, and other infertility factors (16).

Based on the above findings, the authors of this study proposed that adhesion prevention via placement of expanded polytetrafluoroethylene (ePTFE, trade name Gore-Tex®) around affected ovaries during surgical excision of endometriosis may improve fertility. One meta-analysis demonstrated the benefit of preventing post-surgical ovarian adhesions with the placement of ovarian suspension suture in women undergoing laparoscopic surgery for stage III-IV endometriosis (17). Similarly, this study targeted ovarian adhesion prevention due to the adnexa being predominant sites of adhesion reformation in endometriosis. Further, the role of endometriomas in infertility as previously stated supports focus on the ovaries in this study's adhesion prevention strategy. There are multiple commercial barrier products available for prevention of adhesions, including oxidized regenerated cellulose, hyaluronate carboxymethylcellulose, icodextrin, polyethylene glycol (18), and ePTFE. The primary author of this study chose ePTFE due to its proven effectiveness in decreasing pelvic adhesions after uterine myomectomy and other gynecological surgeries (19–22). Pregnancy rates were then analyzed after excision of endometriosis involving endometriomas ≥3 cm with and without ePTFE.

## Material and methods

### Patient selection

This study was reviewed and approved by the Institutional Review Board. Patients who presented for pelvic pain and/or infertility were enrolled from February 2012 to January 2017 at a single tertiary care center. All women were given informed consent regarding ePTFE and had the option of self-selecting for ePTFE placement after their cystectomy and excision surgery for endometriosis. Patients completed preoperative surveys to assess demographic characteristics and obstetrical/surgical history. Postoperative surveys were mailed six months postoperatively and yearly postoperatively for up to four years to assess menstrual history, pain, sexual activity, fertility, and pregnancy. Inclusion criteria consisted of women who had an endometrioma ≥3 cm confirmed by pathology postoperatively, who did not have a hysterectomy at the time of the index surgery, who completed a preoperative survey, who completed postoperative surveys ≥1 year after surgery, and who reported that they were not avoiding pregnancy post-surgery. Not avoiding pregnancy was defined by sexual intercourse without the use of hormonal contraception, barrier methods, withdrawal methods, or fertility awareness to prevent conception. The distinction between not avoiding pregnancy and seeking pregnancy was made as not all women who became pregnant were seeking pregnancy.

### Surgical technique

Surgeries were completed by a single surgeon using an invariable technique of cystectomy for endometriomas, laparoscopic optimal excision (defined as removing by cutting out, as opposed to ablating, all visible lesions—typical and atypical—suspicious of endometriosis wherever found, having looked systematically using near contact laparoscopy) of endometriosis with carbon dioxide laser, and ePTFE placement around each affected ovary if the patient self-selected for this treatment. ePTFE patches were fixed underneath the ovarian reflection with AbsorbaTack™, allowing the ovary to descend down the sidewall prior to being wrapped with the patch and then finally secured with AbsorbaTack™ to the anterior sidewall. The surgeon documented the American Fertility Society (AFS) adhesion score and classified the endometriosis stage according to The American Society for Reproductive Medicine (ASRM) classifications. Removal was done 10–14 days later laparoscopically by application of gentle traction on the ePTFE patch, which allowed the ePTFE patch and AbsorbaTack™ to be detached together.

### Statistical analysis

Continuous variables were expressed as medians and ranges due to lack of normality of the distributions. Categorical variables were expressed as numbers and percentages. Differences in demographic characteristics, obstetrical/surgical history, pregnancy data, and endometriosis surgical characteristics between women receiving ePTFE and women not receiving ePTFE were assessed using chi-square test and Fisher's Exact test for categorical variables. Independent student's t-test or the Mann-Whitney U test was used for continuous variables depending on distribution normality. Pregnancy rates were compared between groups using chi-square test and Fisher's Exact test. Cumulative 4-year pregnancy rates were calculated with survival analysis using lifetables for women receiving ePTFE and women not receiving ePTFE. Comparison of survival curves was made using the Wilcoxon (Gehan) Breslow statistic. A *p*-value of <0.05 was used to denote statistical significance. All analyses were performed using IBM SPSS Statistics version 23.0 for Windows (Armonk, New York).

### Ethical approval

This study was reviewed and approved by the Saint Louis University Institutional Review Board under protocol 20900.

## Results

### Patient inclusion

**Figure 1:**
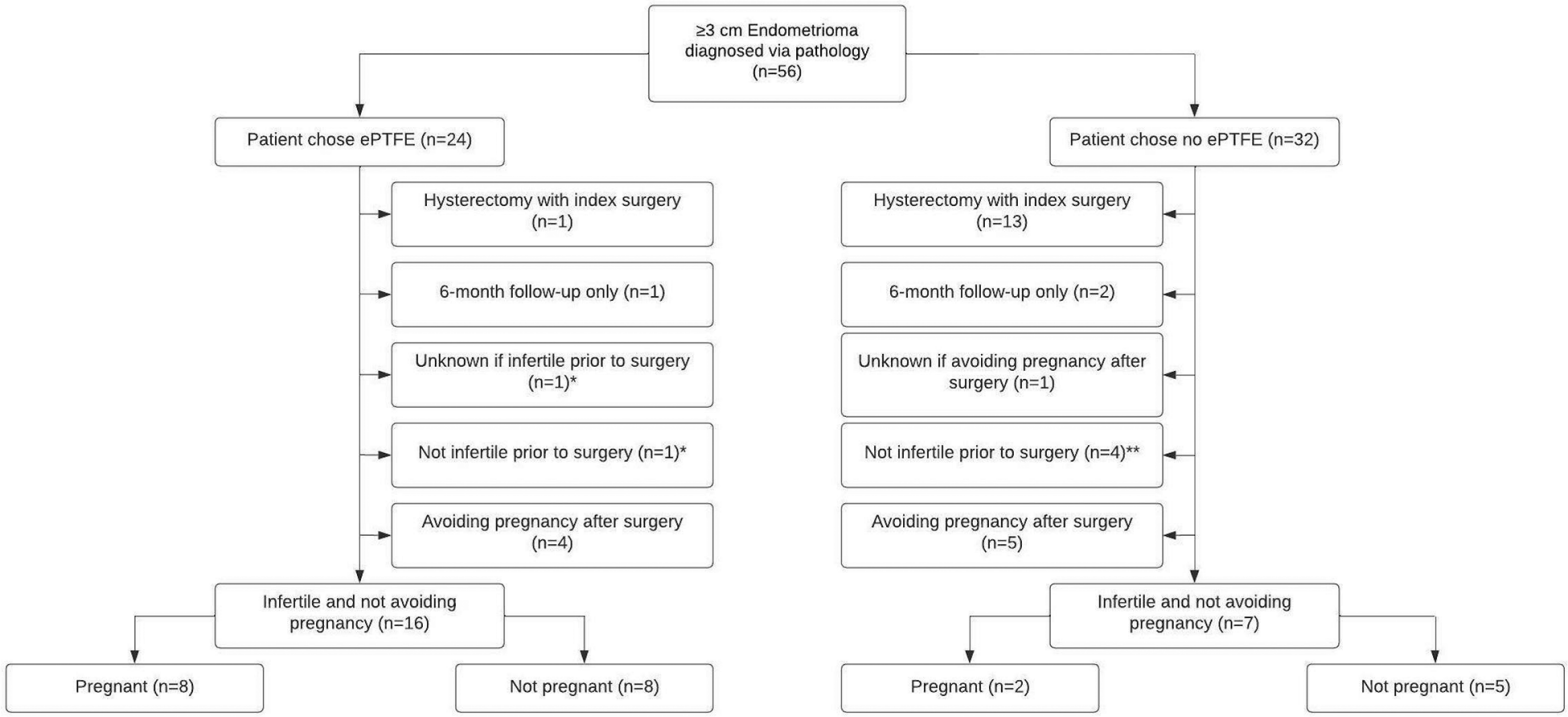
Flowchart of 56 patients who self-selected for ePTFE or no ePTFE after cystectomy of endometriomas ≥3 cm and excision of endometriosis. *Included in the statistical analysis for the ePTFE group. In addition to the 8 out of 16 women who were infertile at the time of the preoperative questionnaire and who were not avoiding pregnancy postoperatively becoming pregnant, 1 woman who was fertile at the time of the preoperative questionnaire and not avoiding pregnancy postoperatively became pregnant, and 1 woman who had unknown fertility status at the time of the preoperative questionnaire and was not avoiding pregnancy postoperatively became pregnant. **Included in the statistical analysis for the no ePTFE group. In addition to the 2 out of 7 women who were infertile at the time of the preoperative questionnaire and who were not avoiding pregnancy postoperatively becoming pregnant, 2 out of 4 women who were fertile at the time of the preoperative questionnaire and not avoiding pregnancy postoperatively became pregnant.

Fifty-six women had an endometrioma ≥3 cm confirmed by pathology (Figure 1). Eighteen women who received ePTFE and 11 women who did not receive ePTFE, who did not have a hysterectomy at time of index surgery, who completed a preoperative survey, who completed postoperative surveys ≥1 year after surgery, and who reported that they were not avoiding pregnancy post-surgery were included in the statistical analysis.

### Demographics and obstetrical/surgical history: overall and by ePTFE group

The median age of the 29 qualifying women was 30 years. The majority of the study population (89.7%) was Caucasian. Approximately 80% of the women had a previous surgery for endometriosis. Twenty-three (82.1%) women reported being infertile at the time of the preoperative questionnaire with a median time of infertility of 48 months and a range of infertility of 12–120 months. All women completed a postoperative survey. The median time from surgery to completion of the postoperative survey was 24 months with a range of 12–70 months. Despite self-selection into treatment groups, the only significant difference in demographic characteristics or obstetrical/surgical history that was found between the ePTFE group and the no ePTFE group was a higher proportion of Caucasian women in the ePTFE group (100.0% vs. 72.7%, *p* < 0.05, Table 1). Although not statistically significant, the high rate of infertility at the time of the preoperative questionnaire in the ePTFE group vs. the no ePTFE group may have clinical significance in that those with infertility may opt to undergo ePTFE placement (94.1% vs. 63.6%, *p* = 0.06). Although not statistically significant, the median time from surgery to the postoperative survey in the ePTFE group (24 months vs. 12 months, *p* = 0.10) may hold relevance in the length of time that ePTFE may continue be beneficial. Although not statistically significant, the median time of infertility at the time of the preoperative questionnaire in the no ePTFE group (84 months vs. 35 months, *p* = 0.15) may be reflective of why these women opted to forego ePTFE placement and elect for hysterectomies.

**Table 1:** Demographic characteristics, obstetrical/surgical history, and pregnancy data for 29 women.

	Gore-Tex®^a^		No Gore-Tex®^b^		*P*-value
Characteristic	(*N* = 18)		(*N* = 11)		
Maternal Age (yr)	29.5 (28–32)		30 (26–32)		0.58
Race					
Caucasian	18	100.0	8	72.7	<0.05
Other	0	0.0	3	27.3	
Smoking					
Never Smoker	12	66.7	10	90.9	
Former Smoker	3	16.7	0	0.0	0.27
Current Smoker	3	16.7	1	9.1	
Previous Surgery for Endometriosis	14	82.4	8	72.7	0.65
Total Number of Pregnancies	0 (0–1.00)		0 (0–0.25)		0.41
Number of Full Term Births (≥37 weeks gestation)	0 (0–0)		0 (0–0)		0.69
Number of Therapeutic Abortions	0 (0–0)		0 (0–0)		0.83
Number of Miscarriages	0 (0–1.00)		0 (0–0.25)		0.59
Number of Living Children	0 (0–0)		0 (0–0)		0.83
Infertile at Time of Preoperative Questionnaire^c^	16	94.1	7	63.6	0.06
If Infertile at Time of Preoperative Questionnaire, How Long? (mo)	35 (24–60)		84 (60–84)		0.15
Completed Postoperative Survey	18	100.0	11	100.0	—
Time from Surgery to Postoperative Survey (mo)	24 (16.5–51.0)		12 (12–24)		0.10
Repeat Surgery for Endometriosis Performed	2	11.1	1	9.1	1.00
Trying to Get Pregnant at Time of Postoperative Survey	17	100.0	11	100.0	—
Became Pregnant Post-Surgery					
Fertile Women at Time of Preoperative Questionnaire					
Yes	1	100.0	2	50.0	1.00
No	0	0.0	2	50.0	
Infertile Women at Time of Preoperative Questionnaire					
Yes	8	50.0	2	28.6	0.41
No	8	50.0	5	71.4	
All Women^d^					
Yes	10	55.6	4	36.4	0.54
No	8	44.4	7	63.6	
Time Trying to Get Pregnant Post-Surgery (mo)^e^	10.5 (6.75–20.25)		7.5 (5.5–26.0)		0.73
Pregnancy Treatment (any)^e^	6	60.0	2	50.0	1.00

Gore-Tex® is the trade name for polytetrafluoroethylene (ePTFE).

Data are presented as medians and interquartile ranges (IQR) or as *n*, %.

^a^ Previous endometriosis surgery, infertile at time of preoperative questionnaire, and trying to get pregnant post-surgery were unknown for one woman.

^b^ Total number of pregnancies, number of full term births, number of therapeutic abortions, number of miscarriages, and number of living children were unknown for one woman.

^c^ Infertile defined as random acts of intercourse for ≥12 months while doing nothing to avoid pregnancy.

^d^ One woman in the Gore-Tex® group who had unknown fertility status at the time of the preoperative questionnaire also became pregnant.

^e^ Calculated for the 10 women in the Gore-Tex® group who were trying to get pregnant post-surgery and who became pregnant post-surgery and for the four women in the no Gore-Tex® group who were trying to get pregnant post-surgery and who became pregnant post-surgery.

### Endometriosis surgical characteristics: overall and by ePTFE group

The median and range of endometriosis stage of the 29 women were 86 (34–146). The median and range of adhesions score were 24 (0–32). Almost all (96.6%) of the women were ASRM Stage IV. In the ePTFE group, 94.4% of women were ASRM Stage IV with the remaining 5.6% being ASRM Stage III. In the no ePTFE group, 100% of women were ASRM Stage IV. Most patients (82.8%) required surgery for dense adhesions involving the bowel/ureter, bladder surgery with suture, ureterolysis, and bowel surgery without resection. Excisions ≥3 cm were present in 69.0% of the left ovary, 65.4% of the right ovary, and 30.8% of both ovaries. Despite self-selection into treatment groups, the ePTFE group and the no ePTFE group were similar in terms of endometriosis stage, adhesions score, and ASRM stage. Although not statistically significant, it is worth noting the proportion of women in the ePTFE group requiring surgery at the highest level of difficulty compared to the proportion of women in the no ePTFE group (16.7% vs. 0.0%, *p* = 0.35, Table 2).

**Table 2:** Endometriosis surgical characteristics for 29 women.

	Gore-Tex®		No Gore-Tex®^a^		*P*-value
Characteristic	(*N* = 18)		(*N* = 11)		
Endometriosis Stage (“points”)	94 (55.0–121.5)		86 (54–96)		0.60
Adhesions Score	24 (11–32)		24 (4–32)		1.00
ASRM Stage					
Stage III (moderate) (16–40)	1	5.6	0	0.0	1.00
Stage IV (severe) (>40)	17	94.4	11	100.0	
Surgical Difficulty					
Stripping of Ovarian Endometriomas; Deep Endometriosis Not Involving the Bowel/Vagina/Ureter/Bladder; Dense Adhesions Not Involving Bowel/Ureter	1	5.6	1	9.1	0.35
Dense Adhesions Involving the Bowel/Ureter; Bladder Surgery Requiring Suture; Ureterolysis; Bowel Surgery Without Resection Without Resection	14	77.8	10	90.9	
Bowel Resection or Ureteral Reimplantation or Anastomosis	3	16.7	0	0.0	
Left Ovary Excision					
Absent	1	5.6	1	9.1	0.40
Superficial	0	0.0	1	9.1	
<3 cm	5	27.8	1	9.1	
≥3 cm	12	66.7	8	72.7	
Right Ovary Excision					
Absent	0	0.0	2	25.0	0.15
Superficial	1	5.6	0	0.0	
<3 cm	4	22.2	2	25.0	
≥3 cm	13	72.2	4	50.0	
Bilateral Ovary Excision ≥3 cm	7	38.9	1	12.5	0.36

Gore-Tex® is the trade name for polytetrafluoroethylene (ePTFE).

Data are presented as medians and interquartile ranges (IQR) or as *n*, %.

^a^ Right ovary excision and bilateral ovary excision ≥3 cm were unknown for three women.

### Pregnancy rates: overall, by ePTFE group, and by ePTFE group/fertility status

The total number of women who became pregnant was 14/29 (48.3%). The rate of pregnancy in the ePTFE group (55.6% vs. 36.4%, *p* = 0.54, Table 1), median time trying to become pregnant post-surgery in the ePTFE group (10.5 months vs. 7.5 months, *p* = 0.73, Table 1), and rate of pregnancy in the ePTFE group for women who were infertile at the time of the preoperative questionnaire (50.0% vs. 28.6%, *p* = 0.41, Table 1) may have clinical significance. There was no statistically significant difference in pregnancy rates between women receiving ePTFE and women not receiving ePTFE for women who were fertile (100.0% vs. 50.0%, *p* = 1.00). However, the pregnancy rate for fertile women in the ePTFE group was based on a single woman who became pregnant.

Based on postoperative surveys, eight women required postoperative assistance to achieve pregnancy with fertility drugs—including letrozole, hCG, progesterone, and clomiphene—and/or IVF. Six of these patients were in the ePTFE group, and two were in the no ePTFE group. All eight women became pregnant.

**Figure 2:**
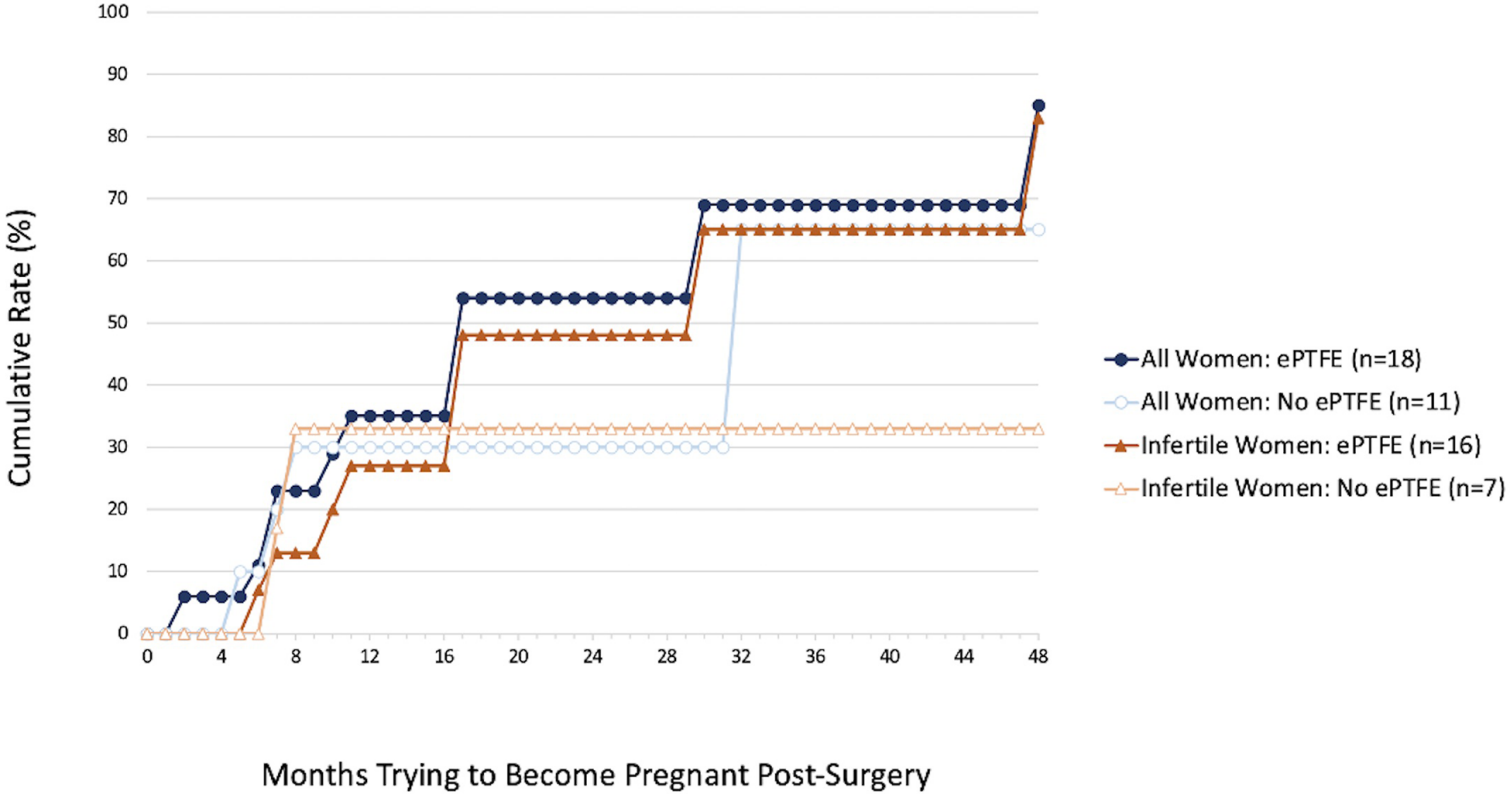
Cumulative 4-year pregnancy rates for all 29 women and 23 women who were infertile at the time of the preoperative questionnaire by whether they received ePTFE after surgery. Higher, although not statistically significant, cumulative 4-year pregnancy rates were found for the ePTFE group compared to the no ePTFE group for all women (85% vs. 65%, *p* = 0.69) and for women who were infertile at the time of the preoperative questionnaire (83% vs. 33%, *p* = 0.89).

The cumulative 4-year pregnancy rates based on lifetable survival curve analysis, which adjusts for differential length of follow-up, also were not significantly different for any period of observation between the ePTFE group and the no ePTFE group. However, it is worth noting the ePTFE group cumulative rates at the 2-year (54% vs. 30%, *p* = 0.71) and 4-year marks (85% vs. 65%, *p* = 0.69, Figure 2) when all women were considered. It is also worth noting the ePTFE cumulative rates at the 2-year (48% vs. 33%, *p* = 0.82), 3-year (65% vs. 33%, *p* = 0.85), and 4-year marks (83% vs. 33%, *p* = 0.89, Figure 2) when women who were infertile at the time of the preoperative questionnaire were considered. The majority of all pregnancies occurred within the first two years post-surgery.

## Discussion

Endometriosis affects up to 50% of women who are infertile (8). Endometriomas are of particular interest in endometriosis research due to their association with diminished ovarian reserve, infertility, and development of adhesions at the adnexa (6, 9). Despite multiple studies demonstrating the ability of ePTFE to reduce adhesion formation in women with endometriosis, there have been no studies that examine adhesions in relation to clinically relevant endpoints, such as pregnancy (4, 7). This study sought to fill this need by exploring the effect of an adhesion prevention strategy that involved ePTFE placement around affected ovaries during cystectomy of endometriomas ≥3 cm and excision of endometriosis on pregnancy rates in infertile women with endometriosis. Laparoscopic excision of endometriosis has been shown to improve fertility (8), and this study aimed to provide additional methods of improving fertility rates for women with endometriosis by targeting the anatomic distortion and recurrence of endometriosis associated with ovarian adhesions.

The pregnancy rates in the ePTFE group seen in this study are promising that ePTFE may improve fertility in women with endometriosis, especially for those who have a history of infertility. 10 of 18 (55.6%) women in the ePTFE group and 4 of 11 (36.4%) women in the no ePTFE group reported pregnancies following cystectomy of endometriomas ≥3 cm and excision of endometriosis. A second analysis was performed to investigate the potential impact of ePTFE on endometriosis-associated infertility by excluding those women who were not infertile at the time of the preoperative questionnaire. 8 of 16 (50.0%) women in the ePTFE group and two of seven (28.6%) women in the no ePTFE group became pregnant.

The cumulative pregnancy rates calculated from lifetable survival curve analysis in the ePTFE group at the 2-year and 4-year marks for all women and at the 2-year, 3-year, and 4-year marks for infertile women, while not statistically significant, are worth noting in that all rates appear promising in the ePTFE group. While most pregnancies occurred within the first two years post-surgery, ePTFE may continue to be beneficial beyond two years. Furthermore, these pregnancy rates were achieved despite a high rate of infertility at the time of the preoperative questionnaire in the ePTFE group (94.1%) when compared to the no ePTFE group (63.6%).

Strengths of this pilot study include homogeneity of surgical procedures afforded by a single surgeon at a single tertiary care institution as well as identification and comparison of many variables including demographic characteristics, obstetrical/surgical history, pregnancy data, and endometriosis surgical characteristics that could have confounded the findings between the ePTFE and the no ePTFE groups. We acknowledge that the former may limit generalizability of the findings which await verification with larger samples in other patient populations with longer periods of follow-up time. The latter found no statistically significant difference on any potential confounding variable between groups, strengthening the validity of the observed elevated pregnancy rates in the ePTFE group.

The small sample sizes of 18 women in the ePTFE group and 11 women in the no ePTFE group who qualified for this pilot study create limitations on the definitive conclusions that can be drawn. A large number of women who declined ePTFE also elected for a hysterectomy, which reduced the size of the no ePTFE group considerably. This study was underpowered to detect significant differences in pregnancy rates due to the small sample size that is inherent in most pilot studies. Nevertheless, the higher pregnancy rates with ePTFE that were obtained in multiple statistical analyses, combined with the reassuring safety profile of ePTFE, indicate that subsequent studies with larger sample sizes that seek to establish the use of ePTFE as a means of treating endometriosis-associated infertility are worth pursuing.

The literature supports that cystectomy of endometriomas and excision of endometriosis with carbon dioxide laser improve fertility outcomes. The majority of the women in our study had ASRM Stage IV endometriosis and still achieved high pregnancy rates. Our pregnancy rates in the ePTFE group, in particular, are comparable to those found in a study where total pregnancy rates, spontaneous or with ART, were similar between women who had their endometriomas managed with either laparoscopic cystectomy (72.2%) or with carbon dioxide laser vaporization (74.4%) (23). Follow-up time from surgery in that study was 13–59 months. The cumulative pregnancy rates of infertile women in the ePTFE group from our study also are similar to those found in another study investigating fertility outcomes after laparoscopic cystectomy in infertile patients with endometriosis (24). Cumulative pregnancy rates from 1 to 5 years post-surgery in that study were 33.9%, 49.2%, 55.9%, 62.7%, and 64.4%. Our study is consistent with these two studies in that the majority of pregnancies occur within the first two years after surgery (23, 24). These results indicate that well-designed studies with larger sample sizes comparing cumulative pregnancy rates, spontaneous or with ART, of women receiving and not receiving ePTFE after cystectomy should be conducted.

This is the first known study to analyze the relationship between adhesion prevention strategy in endometriosis and pregnancy rates. The pregnancy rates with ePTFE use that were observed in this study are promising and suggest that strategies that target adhesion prevention, especially at the adnexa, may improve fertility in women with endometriosis and may be of even greater importance for those with a history of infertility.

## Data Availability

The raw data supporting the conclusions of this article will be made available by the authors, without undue reservation.
